# Predicting Trabecular Bone Stiffness from Clinical Cone-Beam CT and HR-pQCT Data; an In Vitro Study Using Finite Element Analysis

**DOI:** 10.1371/journal.pone.0161101

**Published:** 2016-08-11

**Authors:** Eva Klintström, Benjamin Klintström, Rodrigo Moreno, Torkel B. Brismar, Dieter H. Pahr, Örjan Smedby

**Affiliations:** 1 Department of Medical and Health Science, Division of Radiology, Linköping University, Linköping, Sweden; 2 Center for medical Image Science and Visualization, Linköping University, Linköping, Sweden; 3 KTH Royal Institute of Technology, School of Technology and Health, Huddinge, Stockholm, Sweden; 4 Department of Clinical Science, Intervention and Technology at Karolinska Institutet, Stockholm, Sweden; 5 Department of Radiology, Karolinska University Hospital, Huddinge, Stockholm, Sweden; 6 Institute of Lightweight Design and Structural Biomechanics, TU Wien, Vienna, Austria; Universidad de Zaragoza, SPAIN

## Abstract

Stiffness and shear moduli of human trabecular bone may be analyzed in vivo by finite element (FE) analysis from image data obtained by clinical imaging equipment such as high resolution peripheral quantitative computed tomography (HR-pQCT). In clinical practice today, this is done in the peripheral skeleton like the wrist and heel. In this cadaveric bone study, fourteen bone specimens from the wrist were imaged by two dental cone beam computed tomography (CBCT) devices and one HR-pQCT device as well as by dual energy X-ray absorptiometry (DXA). Histomorphometric measurements from micro-CT data were used as gold standard. The image processing was done with an in-house developed code based on the automated region growing (ARG) algorithm. Evaluation of how well stiffness (Young’s modulus E3) and minimum shear modulus from the 12, 13, or 23 could be predicted from the CBCT and HR-pQCT imaging data was studied and compared to FE analysis from the micro-CT imaging data. Strong correlations were found between the clinical machines and micro-CT regarding trabecular bone structure parameters, such as bone volume over total volume, trabecular thickness, trabecular number and trabecular nodes (varying from 0.79 to 0.96). The two CBCT devices as well as the HR-pQCT showed the ability to predict stiffness and shear, with adjusted R^2^-values between 0.78 and 0.92, based on data derived through our in-house developed code based on the ARG algorithm. These findings indicate that clinically used CBCT may be a feasible method for clinical studies of bone structure and mechanical properties in future osteoporosis research.

## Introduction

Osteoporosis is a major health problem that concerns almost all developed countries and there are big differences in the incidence of hip fractures. Countries like Denmark, Sweden, Norway and Austria have the highest annual hip fracture incidence in women in the world (>500/100,000) [1]. With the increasing longevity of the modern population, there is an increased risk of falls due to impaired balance [2]. The combination of falls and decreased mechanical competence of bone leads to an increase in bone fractures. Bone fractures in elderly, including hip fractures, result in major social and health costs and cause great suffering for the affected individuals.

Changes in the structural and mechanical properties of human bone are correlated with osteoporosis-related fractures, as both the mineral content and the internal trabecular microstructure contribute to bone strength [3–5]. The trabecular structure of bone can, *in vitro*, be evaluated by histomorphometry of bone biopsies or non-invasively by micro-computed tomography (micro-CT). There is good agreement in the literature between these two methods [6]. In patients, the bone mineral density can be assessed and measured by dual energy X-ray absorptiometry (DXA) [7]. The DXA method measures the bone mineral density (BMD) in a specific bone area (g/cm²) and the bone mineral content (BMC) in gram (g). The BMD is a major determinant of bone strength, but many individuals with low impact fractures display BMD values in the osteopenic or normal range [8]. In studies from the 1990’s an alternative device, peripheral Quantitative computed tomography (pQCT), was demonstrated to deliver precise in vivo evaluations of trabecular and cortical density as well as the bone mineral content (BMC) of selected skeletal sites [9]. pQCT was also found to give strong correlations with micro-CT regarding trabecular bone parameters like trabecular number and mean trabecular separation [10]. Another well-described method for visualizing the trabecular bone structure in patients is high-resolution peripheral quantitative computed tomography (HR-pQCT). The HR-pQCT method can be used to evaluate the peripheral skeleton, for example, the heel and the wrist [11–14]. Magnetic resonance imaging (MRI) is also of value for imaging of the trabecular bone structure in patients and is particularly useful as it does not involve radiation to the patient [15, 16]. However, scanning time for MRI is longer, resulting in a greater risk of motion-related imaging artefacts. There is also a risk that magnetic-field dependent susceptibility artefacts may cause overestimation of bone trabeculae [16].

In clinical practice, it would be an advantage to use other clinically available scanners for osteoporotic research of various parts of the human body. Previous *in vitro* studies describe strong correlations between micro-CT and multi-slice CT (MSCT) for bone parameters like bone volume over total volume (BV/TV) [17, 18].

A clinically available modality that may be appropriate for this purpose is dental cone beam computed tomography (CBCT), as the high resolution and the isotropic voxel size (75–400 μm) make the device suitable for imaging small skeletal structures such as the mandible, maxillofacial and temporal bones [19–22]. The dental CBCT technique was first described in 1999 [19] and the use of the technique is rapidly growing. In 2013 there were about 20 manufacturers offering 47 different CBCT devices [23]. There is equipment available for scanning individuals in the standing, sitting and supine positions (http://www.sedentexct.eu/content/comparison-cbct-machines) [23]. The CBCT machines developed to image patients in a supine position can also be used for scanning the peripheral skeleton [24]. CBCT scanners designed for scanning the peripheral skeleton are now available on the market and could potentially be useful for trabecular bone structure analysis and osteoporosis research [25]. Several studies indicate that the bone structure of the mandible can be used for diagnosing osteoporosis and predicting osteoporotic bone fractures [26–29]. We among others have shown that trabecular bone structure parameters obtained by CBCT are strongly correlated to those obtained by micro-CT [17, 30–32]. Imaging of the mandible by CBCT may in the future also be useful for the diagnosis of osteoporosis. It is therefore of interest making a comparison between the clinically already available device, HR-pQCT, and the potentially useful device CBCT using the same segmentation software.

The segmentation process aiming at separating bone from other tissues is a critical step in the analysis [33]. Segmentation can be performed using automated as well as manually applied density thresholds. Unlike MSCT and HR-pQCT, current dental CBCT devices do not provide standardized intensity values (CT-values). CT-values are provided by some scanner manufacturers, but may not be reliable due to influence from factors such as imaging parameters, positioning and the device itself [34, 35]. One way to overcome this is using segmentation methods based on homogeneity thresholding, such as the automated region growing algorithm based on an assessment function (ARG) [36, 37]. This method has in our earlier studies been shown to be appropriate for CBCT data [17, 30]. The ARG algorithm may also be feasible for HR-pQCT data although that equipment does provide standardized CT-values.

Bone biomechanical properties, such as stiffness, shear and strength, can be computed through finite element (FE) analysis based on imaging data [3, 14, 33, 38–43]. Studies have shown strong correlation between bone stiffness and mechanically tested bone strength when computed by FE analysis [44]. The calculated bone biomechanical properties depend on the bone volume fraction and are therefore strongly dependent on the segmentation algorithm [33]. Previous studies have shown that clinical CT machines tend to overestimate the bone volume fraction and the trabecular thickness compared with micro-CT [17, 30, 45]. However, a more relevant question that remains to be answered is whether the morphological measurements computed from images acquired through clinically used CT machines can predict longitudinal stiffness and shear based on micro-CT data.

In view of the incomplete knowledge of the validity of structured parameters from CBCT and of the relationship between such measurements and biomechanical parameters, we have performed a quantitative comparative study of trabecular bone changes associated with osteoporosis using cadaveric radius bone samples. The first aim of this study was to evaluate how closely trabecular bone structure parameters computed on data from different clinical machines correlated with the reference method of micro-CT. The second aim was to evaluate how well stiffness and shear moduli calculated by finite element analysis from micro-CT data could be predicted from the same data.

## Materials and Methods

### Material

Fourteen radius specimens (human wrists) from cadavers were used for the analysis. The specimens were donated for medical research in accordance with the ethical guidelines regulating such donations at University of California, San Francisco. The studied specimens have been used in previous studies [17, 30, 37, 46–48]. The specimens are almost cubic with a side of 12–15 mm and all include slabs of cortical bone.

### Imaging methods and imaging machines

Four imaging techniques were used with five different imaging machines:

- CBCT using the 3D Accuitomo 80 (J. Morita MFg., Kyoto, Japan) in the text referred to as CBCT(A) and the NewTom 5G (QR Verona, Verona, Italy) in the text referred to as CBCT(N)
- HR-pQCT CT using the XtremeCT, (Scanco Medical AG, Brüttisellen, Switzerland)
- DXA data was acquired using the Discovery A S/N 82934, (Hologic Inc, Bedford, MA) with a switched pulse dual-energy at 100 kVp and 140 kVp
- Micro-CT data was acquired with a small desktop CT scanner, the μCT 40 (Scanco Medical AG, Bassersdorf, Switzerland) with isotropic voxel sizes of 20 μm, tube voltage at 70 kVp and tube current of 114 μA

The micro-CT data were used as the gold standard for data comparison. The radiation dose, from the three clinical CT-machines, was given as the computed tomography dose index (CTDI) measured in mGy, reported by the CT manufacturers for each examination. The imaging data parameters for three clinical CT-machines are presented in Table 1.

**Table 1:** Imaging parameters.

Machine	Tube Current	Tube Voltage	Voxel size	FOV	Imaging time	Exposure time	CTDI
	[mA]	[kV]	[μm]	[mm]	[s]	[s]	[mGy]
CBCT(A)	5	85	80	40	17	17	4.9
HR-pQCT	0.9	60 (peak)	82	126	336	-	5.5
CBCT(N)	4.2–4.6	110	75	60	36	7.3	4.1–4.2

CBCT(A)– 3D Accuitomo 80; HR-pQCT–Scanco XtremeCT; CBCT(N)–NewTom 5G

### Specimen preparation

The presence of cortical bone facilitated the orientation for the different analyses. Before imaging the bone samples, fat was removed from the bone samples that were then placed in test tubes filled with water. During imaging in the clinical CT machines, the test tubes were placed in the center of a paraffin cylinder with a diameter of 100 mm. This was to mimic soft tissue and to simulate measurements *in vivo*. To minimize the risk of influence of the large cone-beam angle in the CBCT data, the specimens were carefully centered in the middle of the scanned volumes. Following imaging, bone cubes consisting only of trabecular bone, with a side of approximately 8 mm, were digitally extracted from each data set and used for the analysis. During imaging by using DXA, at protocol subregion Hi-Resolution, the bone cubes were placed in a glass bowl filled with water, with a 2 cm paraffin layer under the specimens, which simulated soft tissue. The BMD measurements were made by choosing rectangular regions of interest (ROI) consisting of only trabecular bone, with sides of approximately 8 mm.

### Image processing

In this study, as in our previous studies [17, 30], we used the ARG algorithm [36] to segment bone from other tissues, or as in the case of this study, from water. To obtain a binary image, the voxels that were identified as bone were assigned the value ʻoneʼ, and all the other voxels became ʻ and. With the ARG method, the separation of tissue structures starts with a very strict homogeneity threshold to define bone, which results in an under-segmented area. The process then repeats with more permissive thresholds until a clear over-segmented region is obtained. The strictest homogeneity threshold is defined as the homogeneity of the original seeds. Those seeds are selected based on the attenuation-value distribution of the entire volume. The most permissive threshold is set as 1.6 times the strictest threshold, which has been found to results in a clearly over-segmented image. Between those thresholds 50 iterations are performed and the iteration where the assessment function reached its minimum was used for the calculations of the following seven bone structure parameters [49].

1. Trabecular nodes (Tb.Nd); measured the number of trabecular intersections per mm^3^
2. Trabecular termini (Tb.Tm); measured the number of free ends of trabeculae per mm^3^
3. Trabecular separation (Tb.Sp); measured the thickness of the spaces between the trabeculae in mm
4. Trabecular spacing (Tb.Sc); measured the distance between the midlines of the trabeculae in mm
5. Trabecular number (Tb.N); measures the number of trabeculae in 1/mm
6. Trabecular thickness (Tb.Th); measures the thickness of the trabecular structures in mm
7. Bone volume over total volume (BV/TV); is measured by dividing the number of voxels classified as bone trabecula by the total number of voxels in the sample.

**Fig 1:**
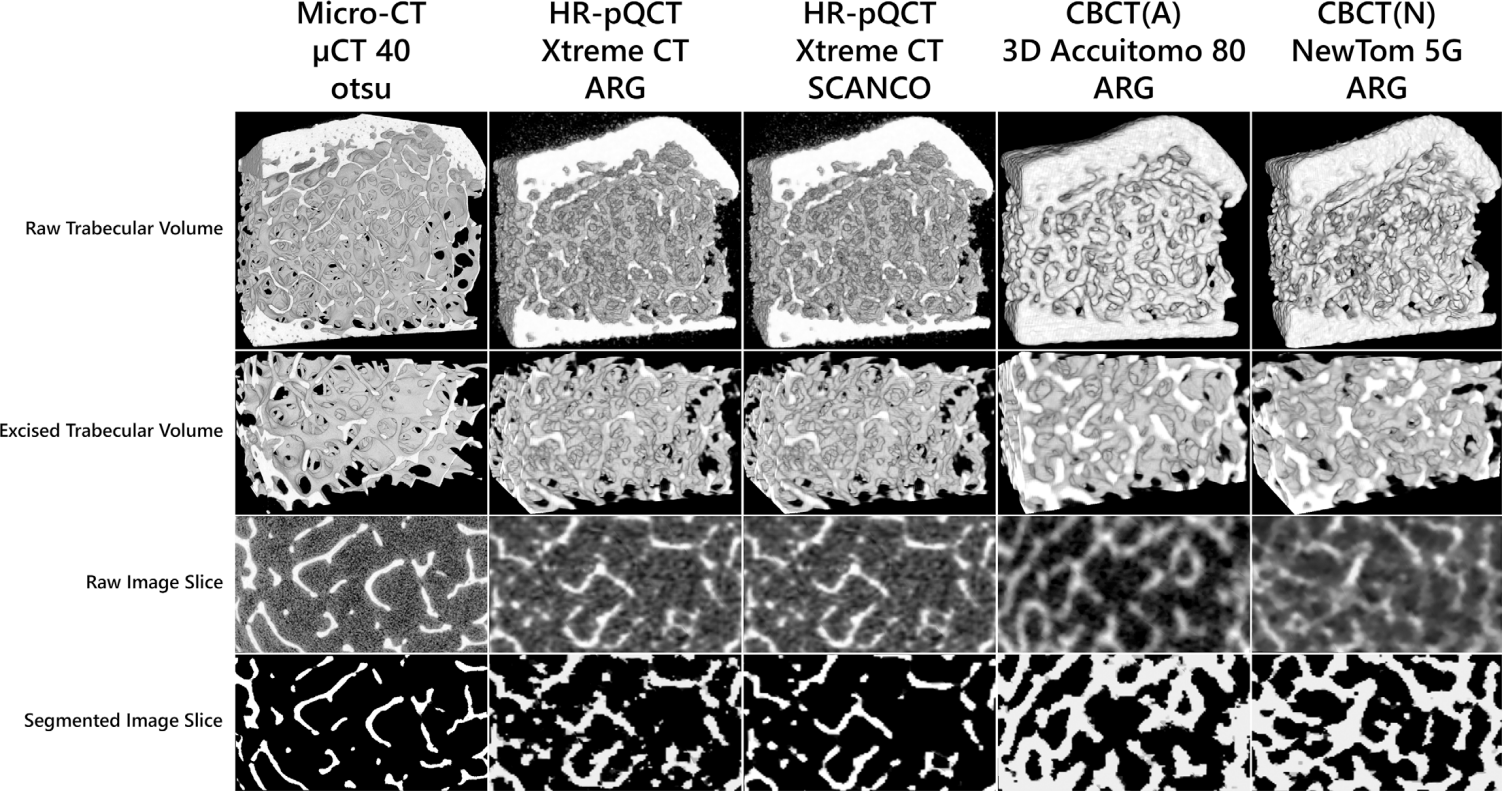
Images of one bone specimen imaged by the different scanners. (A) The same wrist cube imaged by the four different CT machines. Volume renderings of the 3D bone cube are shown in the upper row. Volume renderings of the excised 3D trabecular bone cubes are shown in the second row. Raw images slices are shown in the third row. Segmented images slices are shown in the lower row, where the HR-pQCT data from Xtreme CT is segmented using both an implementation of ARG (automated 3D region algorithm) and an implementation of SCANCO Medical.

**Fig 2:**
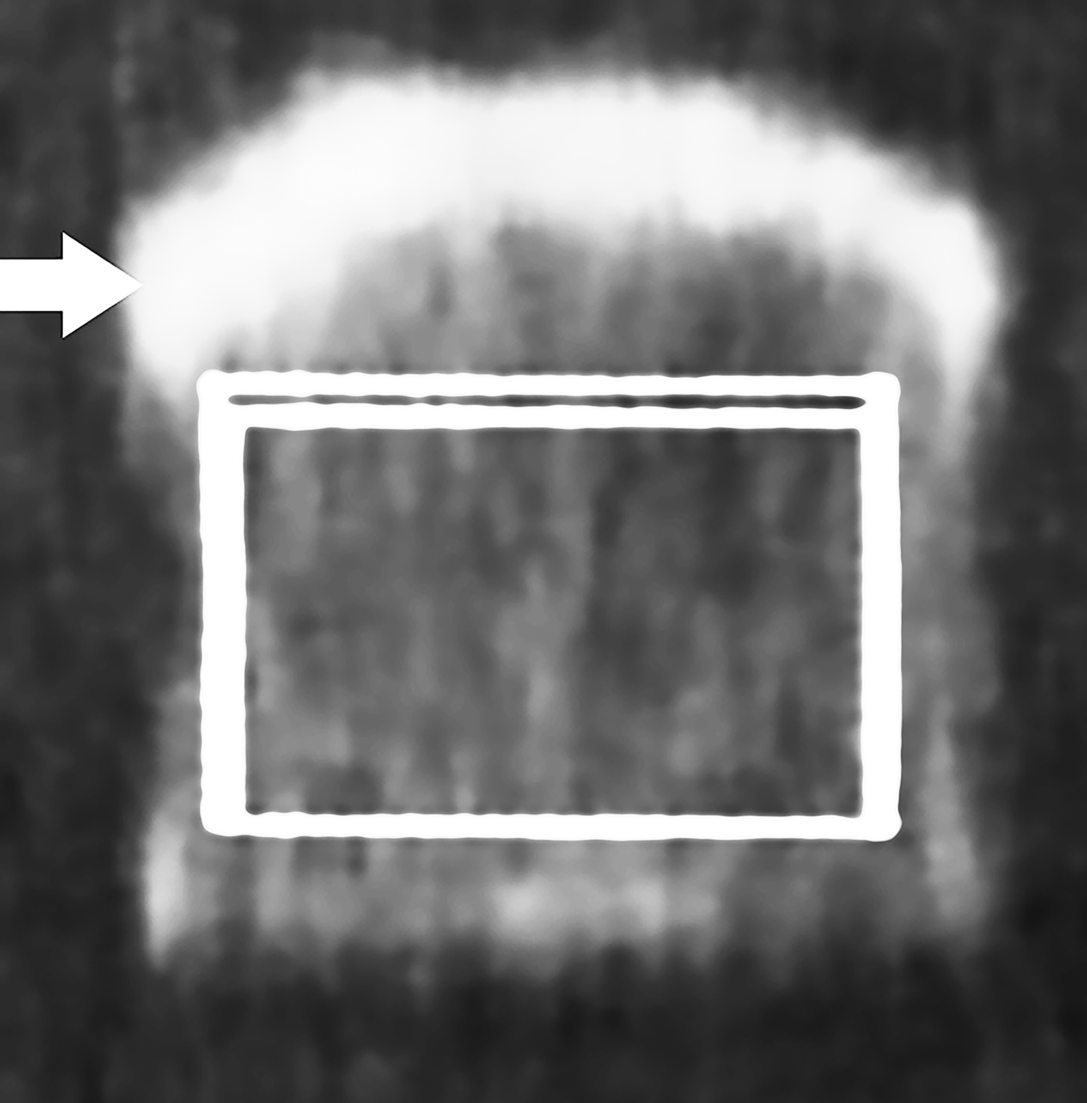
Image of one bone specimen imaged by DXA. The same wrist cube imaged using DXA. The arrow indicates the cortical bone and the white box indicates the volume visualized in Fig 1A, from which the DXA-BMD was calculated.

All parameters were calculated in 3D and in order to remove biases, the same segmentation algorithm was used for all clinical modalities. The four parameters nodes, termini, spacing and number were obtained after skeletonizing the binary image volumes to voxel-wide lines using the method in [50]. 3D renderings of a whole bone sample, 3D renderings of the analyzed trabecular bone volumes as well as raw and segmented images slice from the four analyzed CT-machines can be seen in Fig 1. An image of the same bone cube from the DXA-measurements can be seen in Fig 2.

The parameters were measured and calculated using MATLAB (MathWorks, Natick, MA). The code was developed in-house and calculated on a personal computer (PC) with Intel Core i5 (Intel Santa Clara, CA) at 2.60 GHz, 4 GB random access memory (RAM) and 64-bit operating system. The HR-pQCT data was also segmented using the software from Scanco Medical, which is dedicated for this machine. The micro-CT data was segmented with a method based on gray-level histograms [51]. The measurements for all CT machines, including the contrast-to-noise-ratio (CNR) from the clinical machines, were made in a single run using the same code.

### Analysis of biomechanical properties

**Fig 3:**
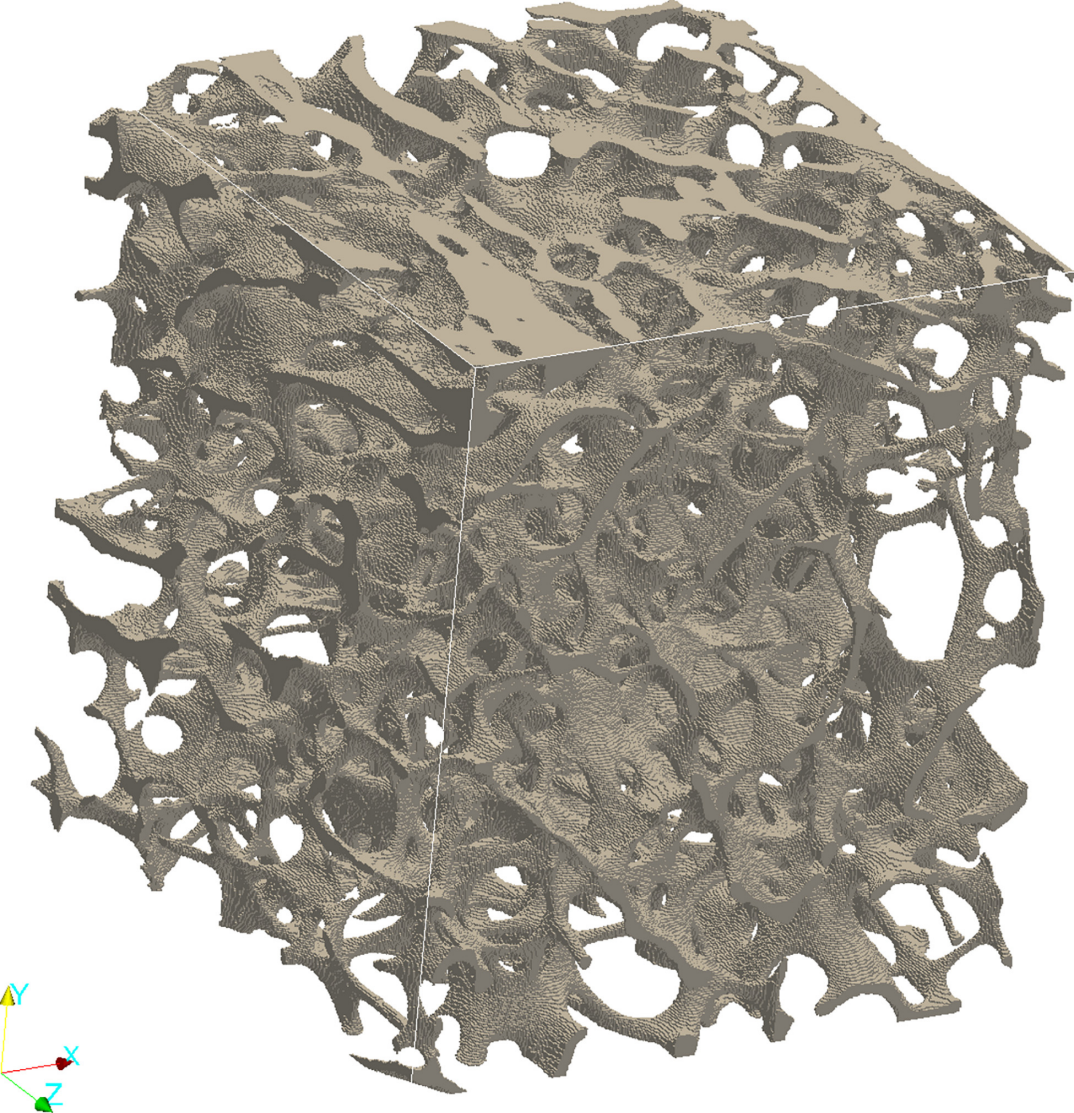
Image of the FE-model of one bone specimen. The FE-model, of the same wrist cube as in Figs 1 and 2, used to calculate Young’s modulus (E1, E2, E3) and minimum shear modulus (G^min^). The model is based on the segmented micro-CT data.

Biomechanical properties of the trabecular bone cubes were derived by finite element (FE) analysis based on the segmented micro-CT data, with sides of 5.3 mm from the center of each trabecular bone cube. A 3D-image of the FE-model for the same bone cube as in Fig 1 and Fig 2 can be seen in Fig 3. Micro-finite element models of the segmented trabecular bone cubes were made by converting image voxels into linear isotropic eight-node hexahedral finite elements. Each element was given a Young’s modulus of 12GPa and a Poisson’s ratio of 0.3 [52, 53]. The apparent elastic properties of the micro-FE models were assessed by performing FE simulations of six independent load cases under kinematic boundary conditions [54]. Testing of the micro-FE models comprised three compressive and three shear tests in which a linear transformation was applied to the surface nodes of the cube. The FE simulations were performed by using Abaqus engineering software (Dassault Systèmes, Paris, France). The full elastic stiffness tensor of each bone cube was computed and the Young’s modulus E1, E2, E3 as well as the minimum shear modulus (G^min^) from the 12, 13, or 23 plane were extracted from the computed stiffness tensor. E3 corresponds to the maximum value, which was aligned to the main loading direction.

### Testing the reproducibility of the methods

To check the reproducibility of the methods used, the bone specimens were scanned twice in the CBCT 3D Accuitomo 80. During this second scanning, it was also possible to check that the status of the bone specimens was unchanged over time. The first scanning was performed two years before and the second scanning just after the scanning in the other clinical machines. The digitally excised trabecular specimens from all clinical CT-machines were then processed and analyzed, as described above.

### Statistical methods

Results are presented as mean values with standard deviations. Data were compared using Pearson correlation with 95% confidence intervals and with linear regression using R^2^-values. Bland Altman analysis was used to asess the reproducibility. Linear and stepwise multiple regression analyses were performed with the IBM SPSS Statistics program. All tables and graphs were created in MS Excel.

## Results

Images of the radius specimens, for studying changes and biomechanical properties associated with osteoporosis, were obtained with four imaging methods including two CBCT devices; 3D Accuitomo 80 referred to as CBCT(A) and NewTom 5G referred to as CBCT(N), one HR-pQCT device; Scanco XtremeCT, one micro-CT device; Scanco μCT 40 and one DXA machine; Discovery A S/N 82934.

The segmentation of HR-pQCT data was made using implementations of two different segmentation methods, both with the in-house-developed code based on the ARG algorithm and with the software dedicated for the XtremeCT device from Scanco Medical. The ARG method worked well also for HR-pQCT data, when compared to the dedicated software, showing somewhat weaker correlations regarding five of the trabecular bone parameters, slightly stronger regarding trabecular nodes and much stronger correlations regarding the parameter trabecular termini (Table 2). In the following data analysis, all results from the HR-pQCT data as well as from the CBCT are based on the in-house developed code.

**Table 2:** Correlations with micro-CT.

Machine	Segmentation	Tb.Nd	Tb.Tm	Tb.Sp	Tb.Sc	Tb.N	Tb.Th	BV/TV
	Method							
CBCT(A)	ARG	0.87	0.79	0.87	**0.94**	**0.94**	**0.92**	**0.96**
		(0.64;0.96)	(0.46;0.93)	(0.63;0.96)	**(0.81;0.98)**	**(0.80;0.98)**	**(0.77;0.98)**	**(0.87;0.99)**
HR-pQCT	ARG	0.79	0.70	0.72	0.73	0.81	0.86	**0.93**
		(0.44;0.93)	(0.27;0.90)	(0.31;0.91)	(0.33;0.91)	(0.50;0.94)	(0.60; 0.95)	**(0.79;0.98)**
HR-pQCT	SCANCO	0.75	-0.27	0.80	0.86	**0.90**	**0.93**	**0.97**
		(0.36;0.92)	(-0.70;0.30)	(0.47;0.93)	(0.61;0.95)	**(0.71;0.97)**	**(0.79;0.98)**	**(0.91;0.99)**
CBCT(N)	ARG	0.79	0.61	0.79	**0.91**	**0.90**	0.86	**0.91**
		(0.45;0.93)	(0.12;0.86)	(0.46;0.93)	**(0.73;0.97)**	**(0.70;0.97)**	(0.62;0.96)	**(0.74;0.97)**

Values are given as Pearson correlation coefficients (r) with 95% confidence limits. **Bold** figures denote values ≥ 0.90. Segmentation methods are an implementation of SCANCO Medical segmentation (SCANCO) and an implementation of Automated Region Growing (ARG)

The two CBCT devices as well as the HR-pQCT machine demonstrated a correlation greater than 0.90 with micro-CT for the bone volume over total volume ratio (BV/TV). Regarding the other bone structure parameters, there were correlations ≥ 0.70 for all machines and all parameters except for termini (Tb.Tm) measured by CBCT(N) that had a correlation of 0.61. CBCT(A) had the strongest correlations with micro-CT for all studied parameters with four parameters showing correlations greater than 0.91 (Table 2).

When predicting stiffness, with simple linear regression, using Young’s modulus E3 as dependent variable, the parameter BV/TV had R^2^-values varying from 0.70 to 0.93 with a p-value lower than 0.001 for all CT-machines (Table 3). The micro-CT device had an R^2^-value of 0.93, showing a strong ability to predict stiffness from this single parameter. The CBCT(A) and the HR-pQCT had R^2^-values ≥ 0.85 and p-values lower than 0.001 for Tb.Tm, indicating the possibility to predict stiffness from this parameter. In addition, when predicting shear, with simple linear regression using the minimum shear modulus (G^min^) from the 12, 13, or 23 as dependent variable, the parameter BV/TV had high R^2^-values, here varying between 0.78 and 0.95 and with p-values lower than 0.001 (Table 4). The micro-CT device had the highest R^2^-value indicating the importance of the parameter BV/TV for predicting the minimum shear using micro-CT. Trabecular termini had R^2^-values ≥ 0.84 for predicting minimum shear using the CBCT(A) and the HR-pQCT devices.

**Table 3:** Results of simple linear regression with stiffness as dependent variable.

Machine	Tb.Nd	Tb.Tm	Tb.Sp	Tb.Sc	Tb.N	Tb.Th	BV/TV
	[1/mm^3^]	[1/mm^3^]	[mm]	[mm]	[1/mm^3^]	[mm]	[%]
CBCT(A)	0.62	0.90	0.75	0.45	0.48	0.05	0.84
	p = 0.001	p < 0.001	p < 0.001	p = 0.008	p = 0.006	p = 0.429	p < 0.001
HR-pQCT	0.85	0.85	0.65	0.62	0.69	0.02	0.71
	p < 0.001	p < 0.001	p = 0.001	p = 0.001	p < 0.001	p = 0.614	p < 0.001
CBCT(N)	0.60	0.67	0.43	0.53	0.52	0.00	0.70
	p = 0.001	p < 0.001	p = 0.011	p = 0.003	p = 0.004	p = 0.993	p < 0.001
Micro-CT	0.55	0.64	0.62	0.44	0.46	0.14	0.93
	p = 0.003	p = 0.001	p = 0.001	p = 0.009	p = 0.007	p = 0.181	p < 0.001

Dependent variable: E3 Youngs´modulus. Values given are R2 values with two-tailed p-values. CBCT(A)– 3D Accuitomo 80; HR-pQCT–Scanco XtremeCT; CBCT(N)–NewTom 5G; micro-CT–μCT 40

**Table 4:** Results of linear regressions with shear as dependent variable.

Machine	Tb.Nd	Tb.Tm	Tb.Sp	Tb.Sc	Tb.N	Tb.Th	BV/TV
	[1/mm^3^]	[1/mm^3^]	[mm]	[mm]	[1/mm^3^]	[mm]	[%]
CBCT(A)	0.51	0.88	0.68	0.36	0.38	0.11	0.86
	p = 0.004	p < 0.001	p < 0.001	p = 0.023	p = 0.019	p = 0.241	p < 0.001
HR-pQCT	0.84	0.84	0.64	0.57	0.63	0.08	0.82
	p < 0.001	p < 0.001	p = 0.001	p = 0.002	p = 0.001	p = 0.337	p < 0.001
CBCT(N)	0.52	0.71	0.35	0.41	0.40	0.02	0.78
	p = 0.003	p < 0.001	p = 0.027	p = 0.014	p = 0.016	p = 0.668	p < 0.001
Micro-CT	0.48	0.68	0.60	0.35	0.37	0.22	0.95
	p = 0.006	p < 0.001	p = 0.001	p = 0.027	p = 0.021	p = 0.094	p < 0.001

Dependent variable: Shear minimum values from the 12,13 or 23 plane. Values given are R2 values with 2-tailed p-values. CBCT(A)– 3D Accuitomo 80; HR-pQCT–Scanco XtremeCT; CBCT(N)–NewTom 5G; micro-CT–μCT 40

All bone structure parameters, except trabecular thickness (Tb.Th), had significance levels ˂0.05 when predicting stiffness and shear with simple linear regression. Trabecular thickness had no significance and very low R^2^-values varying from 0.08 to 0.22 when predicting stiffness and minimum shear (Tables 3 and 4).

**Fig 4:**
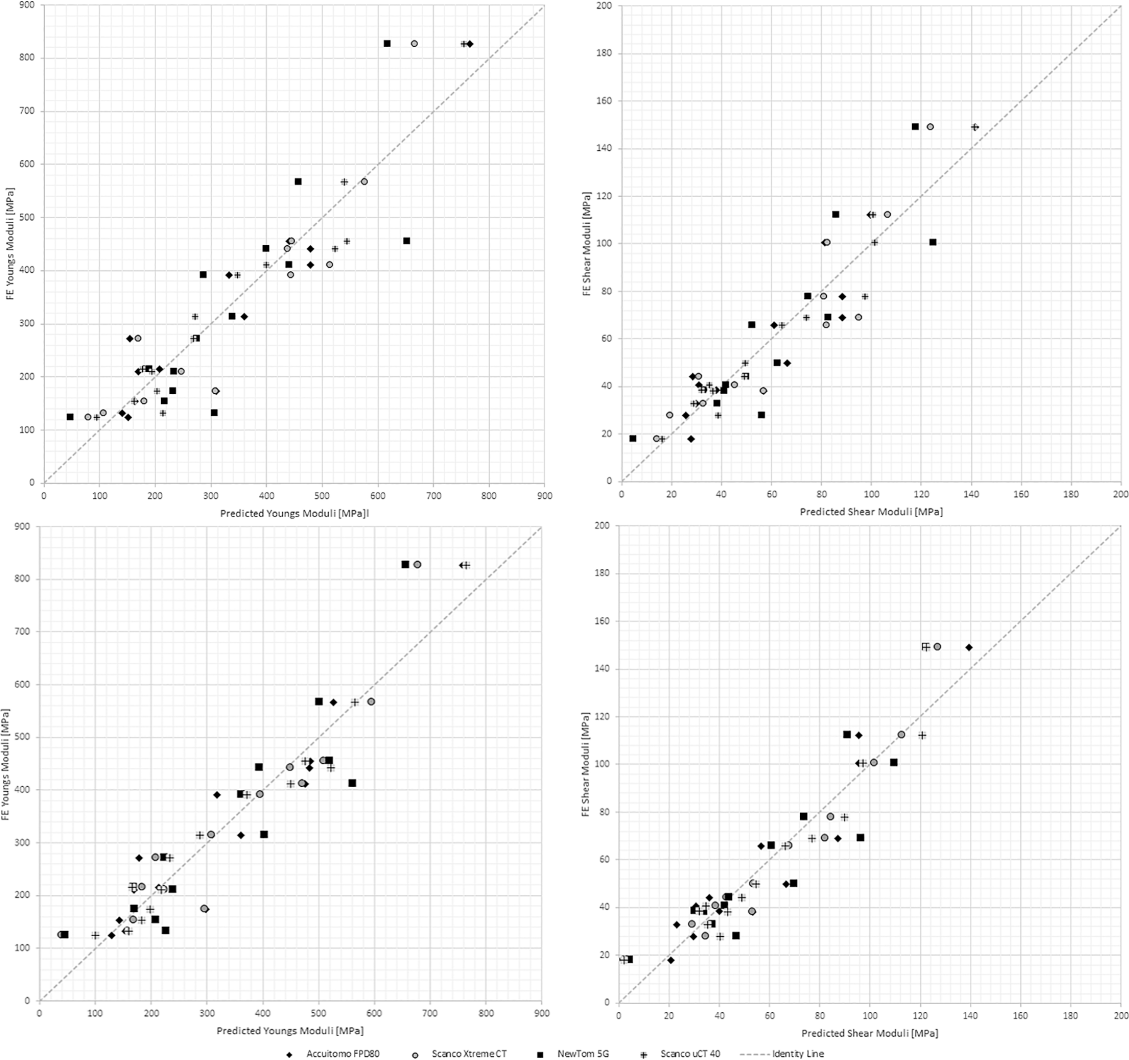
Graphs showing stiffness and shear from micro-CT and predicted stiffness and shear from clinical CT-machines. The stiffness derived by finite element analysis based on micro-CT data as a function of predicted stiffness, calculated as Young’s Modulus *E3* (left panels) and Minimum shear (G^min^) (right panels) based on regression analysis from a single bone parameter (upper panels) or two bone parameters (lower panels). For both stiffness and shear with CBCT 3D Accuitomo 80 and HR-pQCT Xtreme CT data, the single parameter was trabecular termini and the second parameter bone volume over total volume. For both stiffness and shear with CBCT NewTom 5G data, the single parameter was bone volume over total volume and the second parameter trabecular spacing. For stiffness with micro-CT μCT 40 data, the first parameter was bone volume over total volume and the second parameter trabecular thickness. For shear with micro-CT μCT 40 data, the single parameter was bone volume over total volume and the second parameters trabecular spacing.

Both HR-pQCT and the CBCT(A) were highly dependent on the bone parameter Tb.Tm when, through stepwise multiple regression analysis, predicting stiffness with Young´s modulus E3 as dependent variable. The micro-CT and the CBCT(N) were both more dependent on BV/TV. Predicting stiffness, Young’s modulus E3, from two parameters instead of one, did increase the adjusted R^2^-values for the CBCT(N), but had no stronger effect regarding the other machines (Table 5 and Fig 4). In addition, when predicting the shear minimum value, through stepwise multiple regression, the HR-pQCT and the CBCT(A) devices depended on Tb.Tm as a single predictor and added BV/TV as the second predictor. The micro-CT and the CBCT(N) depended on BV/TV as a single predictor and added the parameter trabecular spacing (Tb.Sp) as the second predictor (Table 5 and Fig 4).

**Table 5:** Results [R^2^] of stepwise multiple linear regression with stiffness [E3] and shear [minimum in 12,13 or 23 plane] respectively as dependent variable.

Machine	stiffness [E3]		shear [minimum]	
	Single predictor	Two predictors	Single predictor	Two predictors
	[R^2^]	[Adjusted R^2^]	[R^2^]	[Adjusted R^2^]
CBCT(A)	0.90^a^	0.89^c^	0.88^a^	0.89^c^
HR-pQCT	0.85^a^	0.87^c^	0.84^a^	0.92^c^
CBCT(N)	0.70^b^	0.78^d^	0.78^b^	0.80^d^
Micro-CT	0.92^b^	0.95^e^	0.95^b^	0.90^d^
DXA	0.07		0.11	

Predictors: a) Tb.Tm, b) BV/TV, c) Tb.Tm and BV/TV, d) BV/TV and Tb.Sc, e) BVTV and Tb.Th. CBCT(A)– 3D Accuitomo 80; HR-pQCT–Scanco XtremeCT; CBCT(N)–NewTom 5G; micro-CT–μCT 40; DXA–Discovery A

The bone mineral density (BMD) values from the DXA measurements, measured in g/cm^2^, of the 14 trabecular bone cubes were low and varied between 0.004 and 0.018 g/cm^2^. The BMD was not correlated with the bone volume over total volume ratio measurements from the micro-CT data and gave poor results predicting both stiffness (R^2^ = 0.07) and shear (R^2^ = 0.11) (Table 5).

The CBCT scanners overestimated the mean bone volume over total volume ratio when compared with the reference method, micro-CT (Table 6). The mean thickness of the trabeculae was overestimated by the CBCT devices as well as by the HR-pQCT device. On the other hand, the number of trabecular nodes (Tb.Nd) was clearly underestimated.

**Table 6:** Basic descriptive statistics.

Machine	Segmentation	Tb.Nd [1/mm3]		Tb.Tm [1/mm3]		Tb.Sp [mm]		Tb.Sc [mm]		Tb.N [1/mm3]		Tb.Th [mm]		BV/TV [%]	
	method	Mean	SD	Mean	SD	Mean	SD	Mean	SD	Mean	SD	Mean	SD	Mean	SD
CBCT(A)	ARG	1.48	0.30	1.15	0.27	0.54	0.05	1.07	0.09	0.94	0.08	0.48	0.04	0.42	0.08
HR-pQCT	ARG	1.21	0.39	0.69	0.15	0.78	0.11	1.14	0.15	0.89	0.11	0.30	0.03	0.17	0.06
HR-pQCT	SCANCO	1.26	0.51	0.99	0.24	0.78	0.11	1.05	0.12	0.97	0.11	0.24	0.02	0.12	0.05
CBCT(N)	ARG	1.55	0.25	1.57	0.20	0.59	0.05	1.06	0.07	0.94	0.07	0.44	0.03	0.38	0.05
Micro-CT	otsu	5.32	1.51	0.87	0.25	0.63	0.09	0.85	0.10	1.20	0.14	0.13	0.01	0.10	0.03

Values are given as mean values with standard deviations [SD]. Segmentation methods are an implementation of SCANCO Medical segmentation (SCANCO), an implementation of Automated Region Growing (ARG) and an implementation of otsu-thresholding (otsu). CBCT(A)– 3D Accuitomo 80; HR-pQCT–Scanco XtremeCT; CBCT(N)–NewTom 5G; micro-CT–μCT 40

The radiation dose given from the clinical machines in (CTDI) measured in mGy was rather equal for the both dental CBCT devices despite less than half radiation exposure time for CBCT(N) compared to CBCT(A) (Table 1). The contrast-to-noise ratio (CNR) varied from 6.0 to 10.4, with the highest value for the CBCT(A).

**Fig 5:**
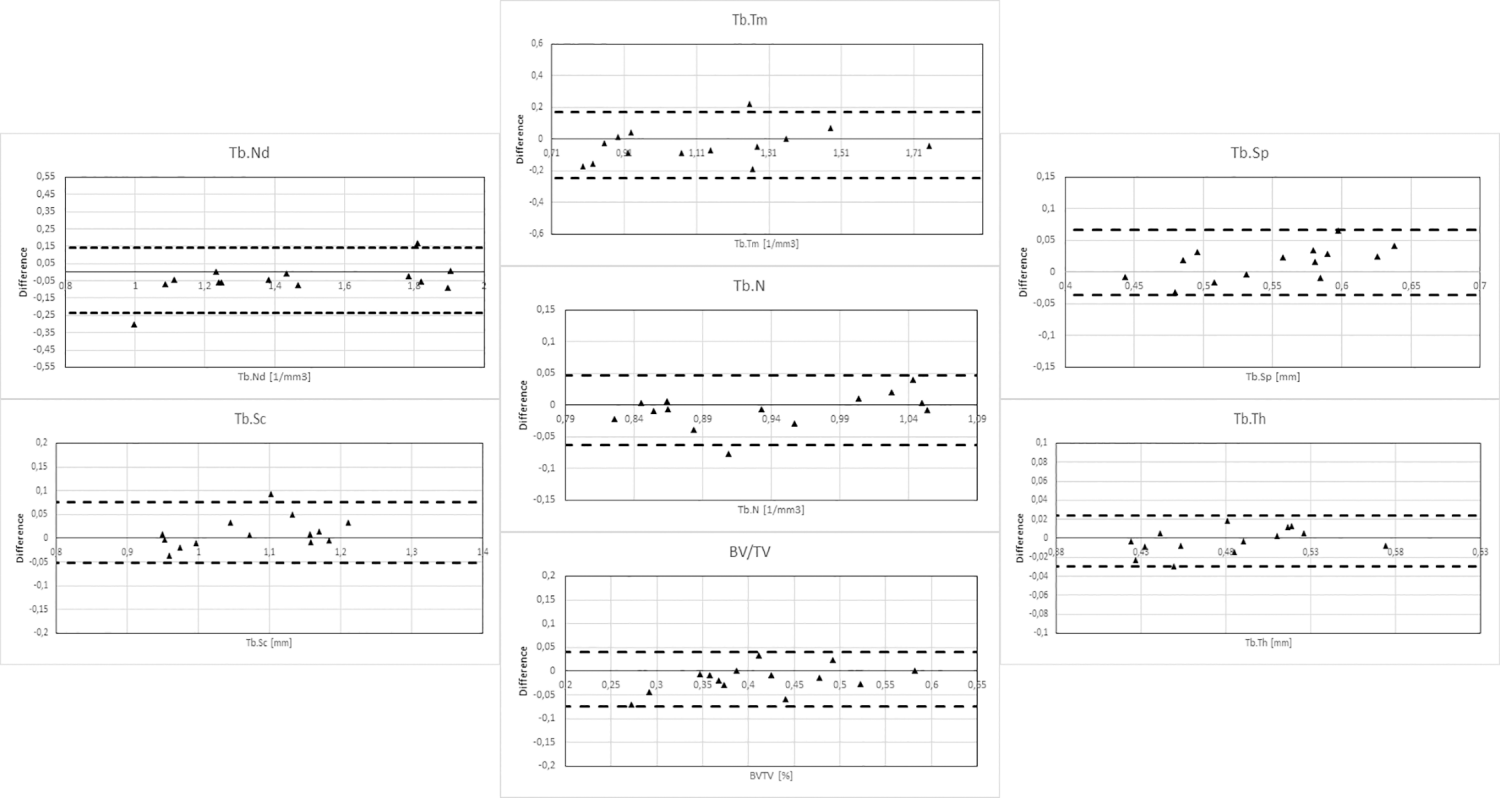
Bland Altman plots for reproducibility of CBCT(A)-data. Bland-Altman analysis of long-term reproducibility of the derived parameters describing trabecular bone histomorphometry. The scans of the bone cubes were made using a CBCT 3D Accuitomo 80 with a time interval of two years.

Regarding the reproducibility of the methods, when the radius bone cubes were repeatedly imaged in CBCT(A) and then processed and analyzed, very good reproducibility was obtained with strong correlation to the reference method micro-CT and without any systematic errors detected (Fig 5).

## Discussion

In this study, we have demonstrated the ability of dental CBCT for predicting trabecular bone stiffness and shear based on micro-CT data. When using the in-house developed code, based on the ARG algorithm, to assess trabecular bone structure parameters, a strong correlation for all studied parameters could be observed between the three studied clinical CT machines (two CBCT and one HR-pQCT device) and the ʻgold standardʼ imaging method micro-CT. The strongest correlations were found for the bone volume over total volume ratio (BV/TV), where all CT machines showed correlation coefficients above 0.90. The two CBCT machines had correlations coefficients of 0.90 or greater with regard to trabecular spacing (Tb.Sc) and trabecular number (Tb.N). The CBCT 3D Accuitomo 80 (CBCT(A)) had the overall strongest correlation coefficients being above 0.91 for four of the seven studied bone structure parameters.

The three studied clinical CT-machines were all able to predict stiffness as the Young’s modulus E3 with acceptable results. The CBCT(A) and the Xtreme CT (HR-pQCT) predicted stiffness with good and rather equal R^2^-values (0.90 and 0.85), which were greater than for the CBCT NewTom 5G (CBCT(N)). The bone structure parameter trabecular termini (Tb.Tm) showed a weaker correlation with μCT than did the other parameters. Despite that, both HR-pQCT and CBCT(A) could predict stiffness and shear well based on Tb.Tm. These high correlations were an unexpected finding that needs to be confirmed by future studies. One should, however, bear in mind that this parameter is strongly influenced by details of the skeletonization algorithm used and need not show the same behavior with a different implementation. This may raise the question whether the algorithm used in this study might be more suitable for measuring Tb.Tm from CBCT and HR-pQCT data than from micro-CT data.

CBCT(N) generally had a weaker correlation to micro-CT than CBCT(A); this was probably due to a difference in exposure parameters. The CBCT(N) imaged a volume cylinder of 6×6 cm while the CBCT(A) imaged a cylinder of 4×4 cm. As the radiation dose, measured as CTDI, was similar for the devices, the exposure per volume was lower for the CBCT(N) resulting in a lower contrast-to-noise-ratio (CNR) affecting the image quality negatively.

In this study, the ability of bone mineral density (BMD), measured by DXA, to predict stiffness and shear was poor. This was probably due to the small size of the cubes, 8 mm in side, resulting in a low BMD (0.004–0.018 g/cm^2^). This density is probably too close to the detection level of the scanner, designed to quantify BMD of the spine and hip, with BMD around 0.8–1 g/cm^2^. An *in vitro* study in rat bone performed to predict cortical bone fracture loads from DXA and dental CBCT found that CBCT imaging had superior predictive value when compared to DXA. In that study, the BMD measured by DXA was about 0.14 g/cm^2^ which is also lower than in ordinary clinical DXA use [55]. DXA is normally only used to analyze the amount of bone and not to assess structure. However, it is possible to analyze the grey-level textural matrix, the trabecular bone-score (TBS). In our study, the evaluation of TBS was not available on the DXA device used. TBS has shown to provide aspects of skeletal structure not reflected by the BMD values [56]. Yet other studies have not been able to show that TBS was superior to areal BMD in predicting vertebral fracture risk [57] or give additive value to BMD when determining bone stiffness [58]. The impact of TBS therefore remains controversial.

At clinical imaging, there is a risk for patient movement artefacts. A short imaging time minimizes this risk. The scanning times in our study for the CBCT devices (17.5–36 s) may cause motion artefacts when imaging patients. To reduce the imaging time, it is possible to perform a 180-degree rotation at nine seconds when using CBCT(A). An earlier study by our group [30] showed that the correlation with micro-CT as well as the CNR was reduced with this lower rotation angle. The imaging time using CBCT(N) can be reduced by speeding up the 360-degree rotation time to 24 s instead of 36 s which reduces the radiation time from 7.3 to 4.5 s. However, there is a risk that this may result in lower CNR, as occurs for CBCT(A) when radiation time is decreased [30]. Therefore, decreasing the scanning time in these dental CBCT devices may not be the ideal solution for reducing risk of motion artefacts when scanning patients in the clinic.

When considering future research studies using dental CBCT there are both disadvantages and advantages to take in account. One advantage is that dental CBCT scanners most often are used for examinations only during office hours. This means there is free capacity for research studies when the scanners are not in clinical use. Both an advantage and a challenge is the possibility of changing the scanning parameters, such as field of view (FOV), tube current and tube voltage, in many ways. This is an important factor to consider since it affects the radiation dose to the patient. [59].

The imaging machines use different imaging planes and require individuals to be in different positions, which may affect imaging motion artefacts. When imaging in the HR-pQCT, the patients are sitting with their limb fixed, while in the CBCT(N) the patients are in the supine position. Both these positions would result in reduced risk of motion artefacts. When imaging in CBCT(A), patients are in the sitting position. Although their heads and cheeks are fixated with straps, this position may increase the risk of motion artefacts. However, a recent clinical study demonstrated that the small middle ear structures could easily be demonstrated by CBCT(A) when used clinically [60]. Further clinical studies are necessary to investigate the possibility of imaging the trabecular bone structure *in vivo*. To image the wrist in living individuals using CBCT(A), a fixation device is required; this is currently being developed by our group.

Another difference to consider is the varying voxel volumes of the different imaging techniques, which causes partial volume effects that may be more or less prominent. They can be expected to affect the results more in vivo than in vitro. A way to reduce the problem of partial volume effects is to replace binary segmentation with direct calculation of the relevant trabecular structure parameters from the gray-scale images. However, to our knowledge, a method for this is available only for Tb.Th [61]. Our plans for future research include implementing gray-scale-based algorithms for a number of structure parameters.

In this study, segmentation data from the three clinical CT-machines were achieved using our in-house developed software; segmentation of HR-pQCT data in many other studies use the software provided by the manufacturer of the imaging machine. In order to evaluate this, the segmentation of the HR-pQCT data was done using both an implementation of the software from the manufacturer and with our ARG-based software. The strong correlation that we detected between HR-pQCT and the gold standard technique of micro-CT for trabecular bone structure parameters, as well as the high R^2^-values predicting stiffness and shear from our segmented data, indicate that our software and analysis may be useful for the evaluation of HR-pQCT data.

There are limitations to this study. The most obvious limitation is the small sample size. However, the specimens used were imaged using several scanning machines and techniques that allowed the study of a number of imaging parameters and computation of several variables [17, 30]. Yet another limitation is the use of cadaveric bone cubes stored for years. However, the rescan of the bone cubes in CBCT(A) and the reanalysis of the imaging data, showed that the radius specimens used in this study were unchanged over time. The fact that the specimens not were surrounded by cortical bone may have an impact on the imaged trabecular bone structures. The micro-CT used in this study, however, could not image such large samples. This kind of cadaveric studies may not reflect conditions in vivo and future clinical studies to support our findings are needed. To be able to include more brands into the analysis, integration into PACS-systems is needed and this is another of our ongoing projects.

When doing research using dental CBCT devices there will be possibilities to study the mandibular bone. There are many studies showing the correlation between mandibular trabecular bone structure and osteoporosis-related fractures. Those studies depend on subjective assessments of the bone structure in panoramic and intra-oral radiographs [26–29, 62]. It would be appealing to carry out studies with less operator-dependent and more automated methods based on CBCT data, as the one used in this study. As the trabecular bone structure plays a major role in supporting dental implants [63] it would also be of great interest to study bone quality assessed by CBCT with the possibility of potentially correlating the bone structure, as well as the numerically calculated stiffness and shear, to dental implant stability.

In conclusion, the strong correlation between CBCT and micro-CT regarding trabecular bone structure parameters as well as the predictive ability of CBCT for bone stiffness and shear derived by finite element analysis based on the μCT data, indicate that CBCT may be a feasible method for future clinical studies and osteoporosis research.

## Supporting Information

S1 File Code_PLoS.zip. This is the code used to segment and analyse the trabecular bone volumes with the structure parameters as a result.
(ZIP)

S2 File Raw_data.xlsx. This is the Raw-data (structure parameters and FEM-analyses).
(XLSX)
